# Effects of Foliar Selenium Application on Oxidative Damage and Photosynthetic Properties of Greenhouse Tomato under Drought Stress

**DOI:** 10.3390/plants13020302

**Published:** 2024-01-19

**Authors:** Jiawen Song, Lang Xin, Fukui Gao, Hao Liu, Xingpeng Wang

**Affiliations:** 1 College of Water Conservancy and Architecture Engineering, Tarim University, Alaer 843300, China; 18329693159@163.com (J.S.); 18942777099@163.com (L.X.); 2 Modern Agricultural Engineering Key Laboratory at Universities of Education Department of Xinjiang Uygur Autonomous Region, Tarim University, Alaer 843300, China; 3 Institute of Farmland Irrigation, Chinese Academy of Agricultural Sciences, Xinxiang 453002, China; gaofukui1207@163.com; 4 Key Laboratory of Tarim Oasis Agriculture, Ministry of Education, Tarim University, Alaer 843300, China; 5 Key Laboratory of Northwest Oasis Water-Saving Agriculture, Ministry of Agriculture and Rural Affairs, Shihezi 832000, China

**Keywords:** selenium, drought stress, enzymatic response, oxidative damage, photosynthesis, tomato

## Abstract

Both drought stress and exogenous selenium (Se) cause changes in plant physiological characteristics, which are key factors affecting crop yield. Although Se is known to be drought-resistant for crops, its internal physiological regulatory mechanisms are not clear. This study analyzed the effects of selenium application (SeA) on antioxidant enzyme activities, osmoregulatory substance contents, and photosynthetic characteristics of greenhouse tomatoes under drought stress and related physiological mechanisms. The results showed that drought stress induced oxidative damage in cells and significantly increased the content of the membrane lipidation product malondialdehyde (MDA) and the osmoregulatory substance proline (*p* < 0.001) compared with the adequate water supply. The proline content of severe drought stress (W1) was 9.7 times higher than that of the adequate water supply (W3), and foliar SeA increased glutathione peroxidase (GSH-PX) activity, and SeA induced different enzymatic reactions in cells under different drought stresses; catalase (CAT) was induced under severe drought stress (*p* < 0.01) and was significantly increased by 32.1% compared with the clear water control, CAT. Peroxidase (POD) was induced under adequate water supply conditions (*p* < 0.01), which was significantly increased by 15.2%, and SeA attenuated cell membrane lipidation, which reduced MDA content by an average of 21.5% compared with the clear water control, and also promoted photosynthesis in the crop. Meanwhile, through the entropy weighting method analysis (TOPSIS) of the indexes, the highest comprehensive evaluation score was obtained for the S5W3, followed by the S2.5W3 treatment. Therefore, this study emphasized the importance of SeA to reduce oxidative damage and enhance photosynthesis under drought stress.

## 1. Introduction

Se is one of the indispensable trace elements for crop growth, and it has an important regulatory role in crops. Moderate SeA can promote the growth and development of plants, improve fruit quality [1], and enhance crop resilience [2]. According to statistics, 72% of China is in Se deficiency or a low Se state [3]; nevertheless, supplementing Se to the human body through dietary supplements will lead to Se toxicity. Plants are the intermediate link in the Se ecological cycle process; using selenium-enriched crop products to supplement selenium to the human body is a safe and efficient method [4]. Therefore, Se supplementation of plants through foliar SeA also has a significant role for humans. Water is one of the factors necessary for crop growth. In China, although water is scarce, the agricultural water consumption is high. Especially for crops with high water demand in the northern region, drought stress has become an unavoidable problem for plant growth. Water deficits will inhibit the accumulation of photosynthesis products, resulting in fruit stunting and yield reduction [5]. Therefore, under the environment of water scarcity in China, it is greatly important to study the regulatory effects of Se on crops.

The tomato is one of the most widely planted and popular crops in the world [6]. Drought stress improves fruit quality but can be accompanied by the inhibition of plant growth, flower and fruit drop, and yield reduction [7,8,9]. Se is involved in the composition of GSH-PX that catalyzes the reduction of toxic peroxides to harmless hydroxyl compounds, thus protecting biofilms from oxidative degradation [9,10]. When a crop is subjected to an adversarial environment, it will be accompanied by damage to the cell membrane due to elevated levels of reactive oxygen species, an increase in cell membrane lipidation products, and the activation of the plant’s own defense system to resist damage [9]. Superoxide dismutase (SOD), POD, and CAT, on the other hand, are important elements of the defense system. The produced free radicals are scavenged by different enzymes in a division of labor. As the first line of defense of the antioxidant system, SOD first scavenges excessive superoxide anion radicals to maintain the physiological activities of the body; meanwhile, the disproportionation reaction that occurs is also gradually generating low concentrations of H_2_O_2_. At this time, POD and CAT work together to scavenge H_2_O_2_ produced by SOD decomposition, and subsequently, POD is mainly responsible for scavenging low concentrations of H_2_O_2_, and CAT scavenges high concentrations of H_2_O_2_ [11]. At the same time, the crop also improves its osmotic adjustment capacity to adapt to prolonged drought stress [12]. It has been shown that Se alleviates abiotic stresses including drought, improves the antioxidant properties of the crop, activates the chloroplast protection mechanism, and mitigates oxidative stress [13,14,15]. High concentrations of Se can be toxic, whereas low concentrations of Se can have a positive effect by maintaining a homeostatic balance between the scavenging systems and reactive oxygen species production, as well as improving photosynthetic properties [16]. SeA can also effectively alleviate salt stress, increase osmotic potential to maintain crop water, increase leaf photosynthetic pigments, and improve photosynthesis (Pn) [17]. Furthermore, moderate SeA helps plant growth while increasing crop selenium content and yield [1]. However, the regulation mechanism of SeA on drought stress needs to be further explored. Our team’s previous research showed that Se increased the Se content and quality of the fruit while maintaining yield stability.

Both drought stress and Se affect crop physiological growth and even yield. Se can induce enzyme synthesis and plays a key role in improving the defense system. Therefore, it is important to explore the regulatory mechanisms of different SeA concentrations on crops under drought stress conditions. In this study, we analyzed the effects of Se and drought stress on antioxidant enzyme activities, osmoregulatory substances, and photosynthesis in crops, explored appropriate Se concentrations to alleviate oxidative damage and promote photosynthesis, and provided a theoretical base for water conservation and drought resistance to ensure yields. Current research on exogenous substances such as melatonin [18] to alleviate oxidative damage in plants is also relatively mature. In the future, attempts can be made to combine Se and other exogenous substances such as melatonin, and it will be important to explore the joint effects of water-saving irrigation and yield stabilization.

## 2. Results

### 2.1. Effect of Se on Oxidative Damage in Tomato

#### 2.1.1. Leaf Malondialdehyde Content

**Figure 1:**
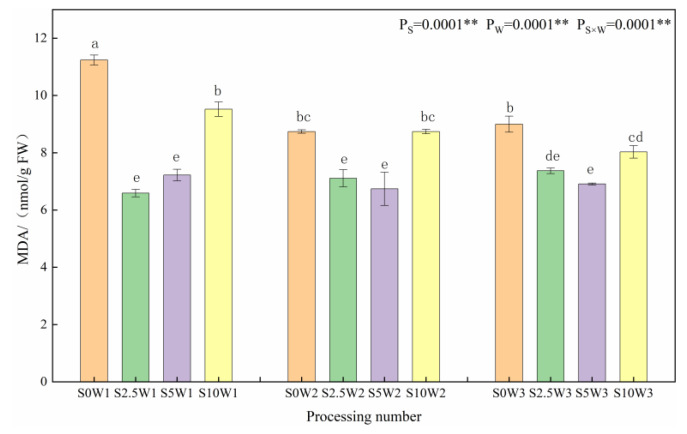
MDA content of leaves in different treatments. MDA: Malondialdehyde. ** indicates significant differences at *p* < 0.01. The means with the same small case letters are statistically non-significant.

As shown in Figure 1, at the same Se spray concentration, MDA content tended to increase with increasing water deficit degree. Under the S0 conditions, W1 significantly increased by 28.6% and 24.9% compared to W2 and W3, respectively, indicating that the S2.5 and S5 leaf MDA was significantly reduced under the S2.5 and S5, and Se alleviated the membrane lipid peroxidation damage. Under the same water deficit condition, the MDA content showed a trend of decreasing and then increasing with the increase in Se concentration, and the MDA content of the three Se concentrations (S2.5, S5, and S10) was lower than that of the control (S0) under the same moisture, with an average reduction of 21.5%. Under severe drought stress (W1), the MDA compared with the S0 was significantly reduced by 41.4%, 35.8%, and 15.3% in the S2.5, S5, and S10, respectively; under mild drought stress (W2) and sufficient water supply (W3) conditions, both the S2.5 and S5 significantly reduced the MDA content compared with the S0, whereas the reduction in MDA content was significantly reduced in the S10. It indicated that too large a concentration of SeA did not play a role in cell membrane oxidation, and the spraying of exogenous Se at low and medium concentrations could significantly reduce MDA content and mitigate the cellular damage by plasma membrane peroxidation.

#### 2.1.2. Leaf Glutathione Peroxidase Activity

**Figure 2:**
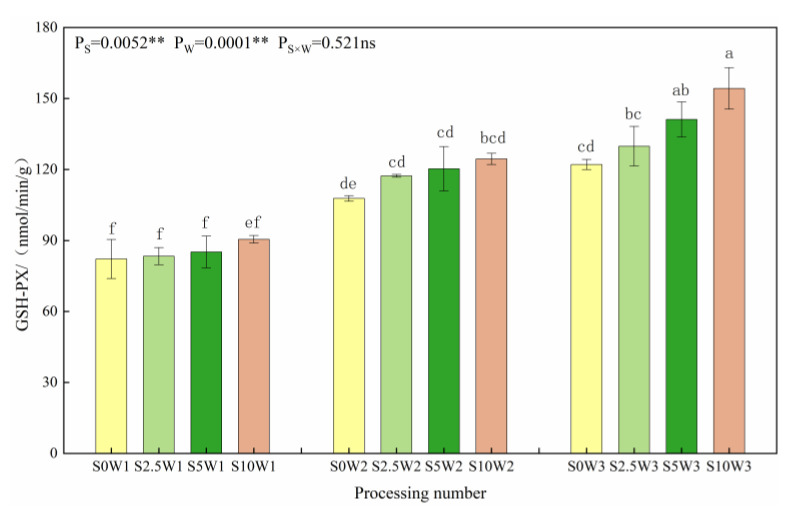
GSH-PX activity of leaves under different treatments. Where “ns” indicates means not significant (*p* > 0.05), ** indicates significant differences at *p* < 0.01. The means with the same small case letters are statistically non-significant.

As can be seen in Figure 2. Under the same Se concentration, the GSH-PX activity increased with a decreasing degree of water deficit, and the average GSH-PX activity increased by 37.7% and 60.4% in W2 and W3, respectively, compared with W1. Under the same water deficit condition, the GSH-PX activity tended to increase gradually with the increase in Se concentration, and all three Se conditions (S2.5, S5, and S10) were higher than the control (S0), with an average increase of 11.8%, and there was no significant difference in GSH-PX activity between different Se concentration conditions under both mild and severe drought stress; under the condition of sufficient water supply, the GSH-PX activity increased with increasing Se concentration, which was significantly increased by 6.3%, 15.6%, and 26.3% in the S2.5, S5, and S10, respectively, compared with the S0. It indicated that SeA increased GSH-PX activity, especially spraying medium to high concentrations of Se under adequate water supply conditions, which significantly increased GSH-PX activity.

#### 2.1.3. Leaf Superoxide Dismutase, Peroxidase, and Catalase Activities

**Figure 3:**
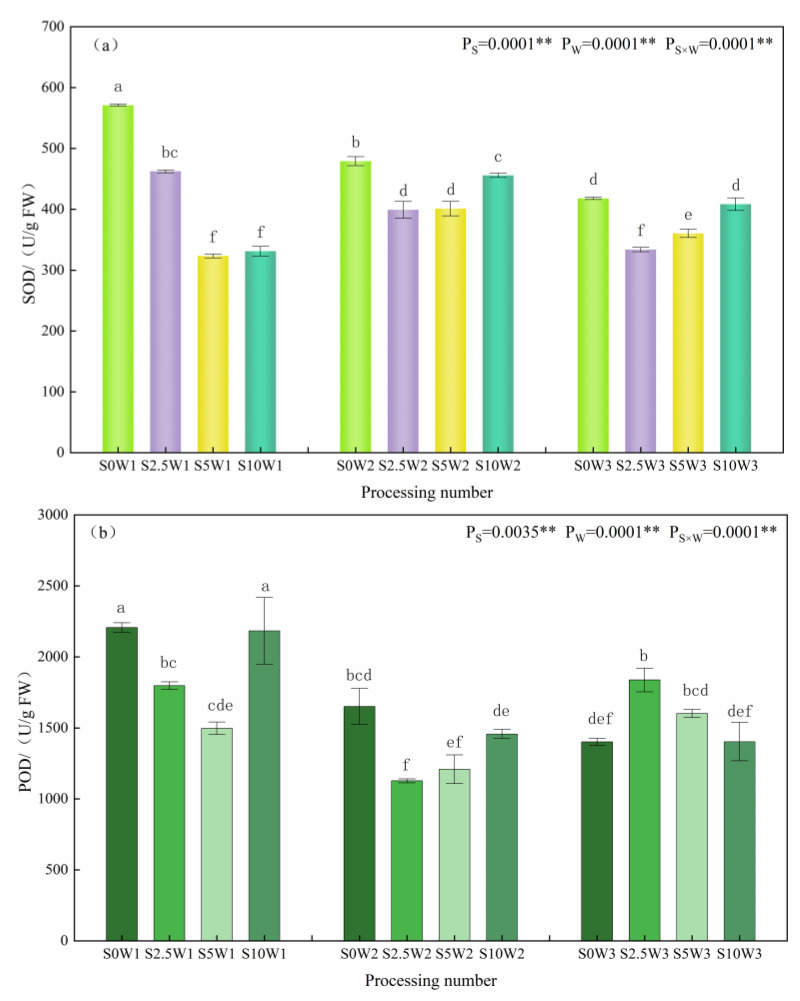

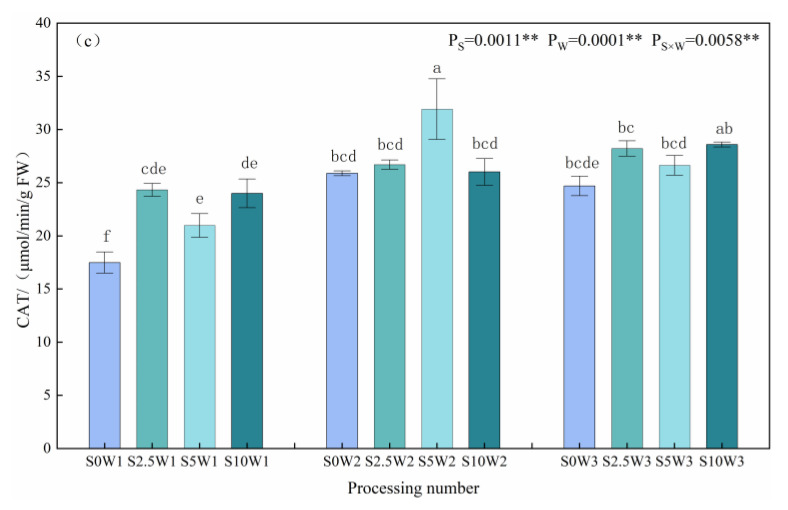
SOD (**a**), POD (**b**), and CAT (**c**) activities of leaves in different treatments. ** indicates significant differences at *p* < 0.01. The means with the same small case letters are statistically non-significant.

Changes in leaf SOD, POD, and CAT activities are given in Figure 3a–c. The effects of Se and soil water deficits, as well as their interaction, on SOD, POD, and CAT activities of leaves reached highly significant levels (*p* < 0.01). Under the S0 condition, SOD activity significantly increased with the increasing degree of water deficit, and W1 and W2 showed significant increases of 36.6% and 14.7%, respectively, compared with W3. Under the same water deficit condition, with the increase in Se concentration, SOD activity showed a trend of decreasing and then increasing, and all of them were lower than that of the control S0, with an average decrease of 26.1% for the three Se conditions (S2.5, S5, and S10), and in the condition of severe drought stress (W1), the S2.5 and S5 were significantly reduced by 19.0% and 43.4%, respectively, compared with the S0. Under mild drought (W2) and sufficient water supply (W3) conditions, the pattern of change in SOD activity was S2.5 < S5 < S10 < S0, in which the S2.5, S5, and S10 under sufficient water supply reduced by 20.1%, 14.0%, and 2.3%, respectively, compared with the S0. An analysis of variance (ANOVA) showed an interaction between Se and soil water deficit, in which SOD activity decreased under severe drought stress at elevated spray concentrations, whereas Se at low and medium concentrations significantly reduced SOD activity under mild drought stress and full water supply conditions.

As far as leaf POD activity was concerned, under the S0 condition, POD activity increased with the increase in water deficit degree, which was significantly increased by 57.4% and 17.7% in the S0W1 and S0W2, respectively, compared with the S0W3, and the pattern of change of POD activity was similar between the S10 and S0, which indicated that the POD activity increased with the increase in water deficit degree, and the change rule of the spraying of high concentration Se was consistent with the control (S0), which may not play a role in alleviating the drought stress of the crop. Under severe drought stress, with the increase in Se concentration, POD activity showed a trend of decreasing and then increasing; the S2.5 and S5 were significantly reduced by 18.5% and 32.1%, respectively, compared with the S0, and the S5 had the smallest POD activity; under mild drought stress, the S2.5 and S5 were significantly reduced by 31.8% and 26.8%, respectively, compared with the S0, and the S2.5 had the smallest POD activity; while in full drought stress, the S2.5 had the largest POD activity. While under the condition of sufficient water supply, the change rule of the POD activity was opposite to that of the two drought degree stresses, and the POD activity of the three Se concentrations (S2.5, S5, and S10) was higher than that of the control (S0), and decreased gradually with the increase in the concentration of Se; the POD activity of the S2.5 was the largest, followed by the S5 and S10 in that order, which significantly increased by 31.1%, 14.3%, 14.3%, and 0.2%, respectively, compared with the S0. Meanwhile, the ANOVA results showed that there was an interaction between Se and soil water deficit, in which low and medium concentrations of Se sprayed under drought stress significantly decreased POD activity under the same moisture level, while low and medium concentrations of exogenous Se sprayed under adequate water supply significantly increased POD activity, probably due to the difference in the amount of H_2_O_2_ produced under different levels of drought stress.

As far as leaf CAT activity was concerned, under the S0 condition, the CAT activities of W2 and W3 were significantly increased by 48.0% and 41.2%, compared with W1; under the three Se (S2.5, S5, and S10) sprayed conditions, the CAT activities of W2 and W3 were increased by 22.1% and 20.4%, respectively, compared with W1, which indicated that Se sprayed increased the CAT activity of W1. Under the same water deficit condition, the CAT activities of the three Se conditions (S2.5, S5, and S10) were higher than that of the control (S0) under the same moisture, with an average increase of 16.3%. Especially under severe drought stress (W1), the three Se conditions (S2.5, S5, and S10) reached a significant level compared with the S0 (*p* < 0.05), with the S2.5, S5, and S10 significantly increasing by 39.1%, 20.0%, and 37.2%, respectively; under mild drought stress (W2), the S5W2 had the greatest CAT activity, which was significantly increased by 23.3% compared with the S0W2. In conclusion, heavy drought stress significantly reduced leaf CAT activity, SeA increased leaf CAT activity under heavy drought stress, and Se at medium concentration significantly increased CAT activity under mild drought stress.

The present study showed that Se reduced leaf MDA content and alleviated the oxidative damage of drought stress on plants. Under severe drought stress, both SOD activity and POD activity showed a tendency to decrease and then increase with the increase in Se concentration, and the spraying concentration of 2.5–5 mg·L^−1^ decreased SOD and POD activities, while increasing CAT activity; compared with the S0, SOD and POD activities of the S2.5 were significantly reduced by 19.0% and 18.5%, respectively, while CAT activity was significantly increased by 39.1% in the S2.5. The SOD and POD activities of S5 were significantly reduced by 43.4% and 32.1%, respectively, and CAT activity was significantly increased by 20.0%. Under adequate water supply, spraying low and medium concentrations of Se reduced leaf SOD activity by 20.1% and 13.8% in the S2.5 and S5, respectively, but increased leaf POD and CAT activities, with an increase in POD by 31.0% and 14.3%, and an increase in CAT by 14.3% and 7.9%, respectively, in the S2.5 and S5, compared with the S0. The effects of Se on various enzymes were different under different degrees of water deficit, which may be due to the different levels of reactive oxygen species in plants with different degrees of drought stress, and the different contents of H_2_O_2_, O^2−^, etc. in plants, and the antioxidant enzymes induced were different [19,20]. 

In the case of abiotic stress, SOD was the primary scavenger of free radicals, while POD scavenged the low concentration of H_2_O_2_, and CAT scavenged high concentrations of H_2_O_2_. Therefore, in this study, the decrease in SOD activity after foliar Se was mainly due to the fact that SOD scavenges free radicals while a disproportionation reaction occurs, leading to an increase in the accumulation of H_2_O_2_ content, which scavenges high concentrations of free radicals, CAT, thus reducing the need for scavenging ROS under drought stress and decreasing the activity of SOD; however, the POD increased under fully watered condition, and this was due to the fact that POD scavenges low concentrations of free radicals. Under the condition of adequate water supply, the amount of ROS released was low, and POD was required to scavenge them, so POD activity was increased.

### 2.2. Effect of Se on Osmoregulatory Substances in Leaves

#### 2.2.1. Leaf Proline Content

**Figure 4:**
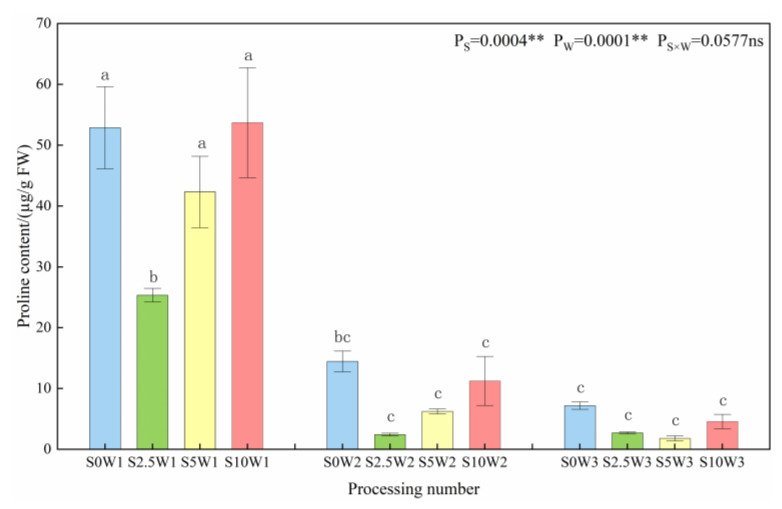
Proline content of leaves in different treatments. Where “ns” indicates means not significant (*p* > 0.05), ** indicates significant differences at *p* < 0.01. The means with the same small case letters are statistically non-significant.

Both Se and soil water deficit had highly significant effects on the proline content of leaves (*p* < 0.01) (Figure 4). At the same Se concentration, the proline content significantly increased with the increasing degree of water deficit, with W1 having 4.1 times and 9.7 times higher proline content than W2 and W3. Under the same water deficit condition, the proline content increased with the increase in Se concentration, and the three Se conditions (S2.5, S5, and S10) decreased by an average of 32.7% compared with the S0, and the proline content of the S2.5 was the smallest under both types of drought stress; it was significantly reduced by 52.0% under severe drought stress compared with the S0, and it reduced by 83.0%. In conclusion, the water deficit significantly affected the proline content, especially under severe drought stress conditions; in contrast, Se reduced the proline content, and the largest decrease in proline content was observed when Se was sprayed at a concentration of 2.5 mg·L^−1^, which indicated that Se alleviated drought stress, and consequently reduced the osmotic adjustment ability of the crop.

#### 2.2.2. Leaf Soluble Sugar Content

**Figure 5:**
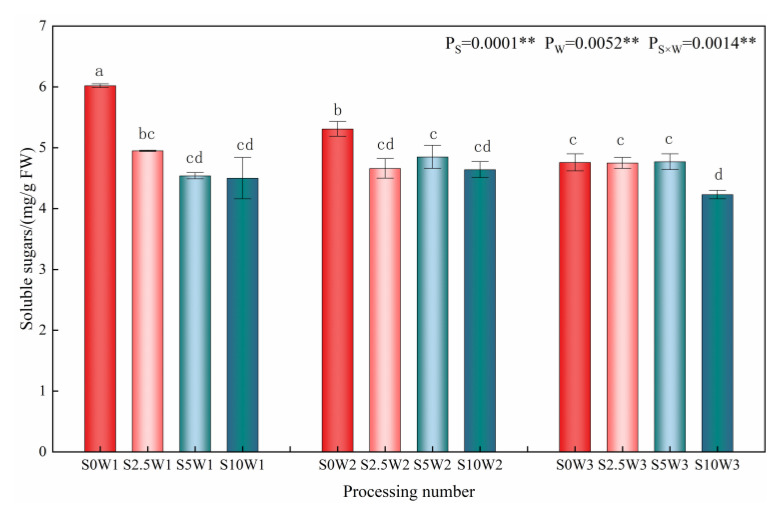
Soluble sugar content of leaves in different treatments. ** indicates significant differences at *p* < 0.01. The means with the same small case letters are statistically non-significant.

Changes in the leaf soluble sugar content are given in Figure 5, where the effects of Se and soil water deficit on leaf soluble sugar content reached highly significant levels (*p* < 0.01). Under the S0 condition, the soluble sugar content increased significantly with the increasing degree of water deficit, and there were no significant differences among the three Se conditions (S2.5, S5, and S10). Under severe drought stress (W1), the soluble sugar content of the three Se conditions (S2.5, S5, and S10) decreased by 17.8%, 24.6%, and 25.2%, respectively, compared with the S0; under mild drought stress (W2), the soluble sugar content of the S2.5, S5, and S10 was reduced by an average of 11.2% compared with that of the S0; under sufficient water supply (W3) conditions, there was no significant difference between treatments. The results of the ANOVA showed that Se interacted with soil water deficit, and Se under drought stress significantly reduced soluble sugar content at the same moisture level, and the greater the degree of drought, the greater the effect of Se on the soluble sugar content.

#### 2.2.3. Leaf Soluble Protein Content

**Figure 6:**
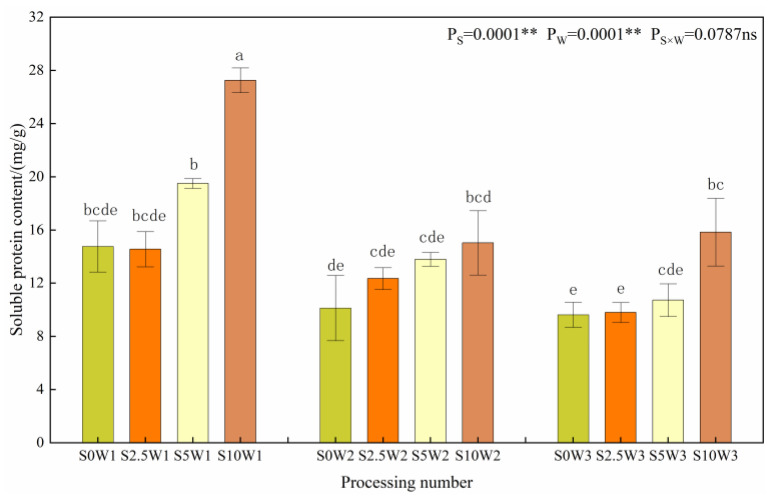
Soluble protein content of leaves in different treatments. Where “ns” indicates means not significant (*p* > 0.05), ** indicates significant differences at *p* < 0.01. The means with the same small case letters are statistically non-significant.

Figure 6 shows the changes in the leaf soluble protein content. The effects of Se and water deficit on leaf soluble protein content both reached highly significant levels (*p* < 0.01). Under the same Se spraying concentration, the soluble protein content gradually increased with increasing degree of water deficit, and compared with W3, the soluble protein content of W1 and W2 significantly increased by 65.4% and 11.6%, respectively, with no significant difference between W2 and W3. Under the same water deficit condition, the soluble protein content had a tendency to increase gradually with the increase in Se concentration, and the soluble protein content of the three Se conditions (S2.5, S5, and S10) increased significantly by 6.3%, 27.5%, and 68.3%, respectively, compared with the S0. Both drought stress and Se tended to promote soluble protein content.

### 2.3. Effect of Se on Photosynthetic Characteristics of Tomato

#### 2.3.1. Leaf Photosynthesis

From Table 1, it can be seen that soil water deficit had a highly significant effect on leaf Pn, Gs, Ci, and Tr (*p* < 0.01), and Se highly significantly affected leaf Pn, Gs, and Tr (*p* < 0.01) but had no significant effect on Ci, and the interaction between Se and soil water deficit had a significant effect on leaf Pn, Gs, and Tr (*p* < 0.05).

At the same Se concentration, leaf Pn, Gs, Ci, and Tr were significantly increased with decreasing water deficit degree, and leaf Pn was significantly increased by 61.7% and 80.8% in W2 and W3, respectively, compared with W1, and Gs, Ci, and Tr were 3.7 times, 5.3 times, and 1.5 times higher in W2 than in W1, and Gs, Ci, and Tr were 5.2 times, 5.7 times, and 2.3 times higher in W3 than in W1, respectively. Under the same water deficit conditions, with the increase in Se concentration, the leaf Pn showed a trend of increasing first and then decreasing, and the change rule of leaf Pn among the three Se conditions (S2.5, S5, and S10) was S5 > S2.5 > S10. Under severe drought stress, the leaf Pn of the S2.5W1 and S5W1 increased by 12.3% and 18.1%, respectively, compared with that of the S0W1; under mild drought stress, leaf Pn was significantly increased by 14.0%, 24.8%, and 3.9% in the S0W1, S2.5W2, S5W2, and S10W2, respectively, compared with that in the S0W2; under sufficient water supply condition, leaf Pn was significantly increased by 10.8% and 12.4% in the S2.5W3 and S5W3, respectively, compared with the S0W3. Leaf Gs decreased gradually with the increase in the concentration of Se sprays (except for S2.5W1), with the S2.5 having the largest leaf Gs, and under adequate water supply conditions, the S2.5W3 significantly increased leaf Gs by 47.6% compared with that of the S0W3; under mild drought stress, the S2.5W2, S5W2, and S10W2 all significantly increased leaf Tr compared with that of the S0W2 by an average of 29.1%.

#### 2.3.2. Relative Amount of Chlorophyll in Leaves

**Figure 7:**
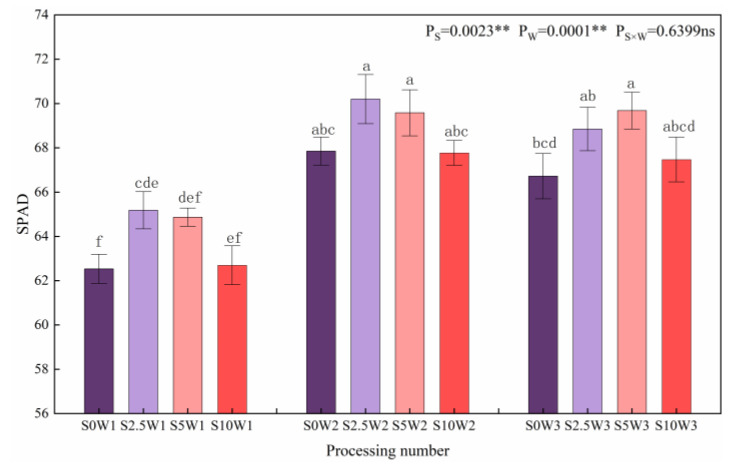
SPAD of leaves in different treatments. Where “ns” indicates means not significant (*p* > 0.05), ** indicates significant differences at *p* < 0.01. The means with the same small case letters are statistically non-significant.

As shown in Figure 7, both soil water deficit and Se had significant effects (*p* < 0.01) on the relative amount of chlorophyll (SPAD) of leaves, and under the same Se concentrations, SPAD showed a trend of increasing and then leveling off with the decreasing degree of water deficit, with W2 and W3 showing a significant increase in SPAD by 7.9% and 6.8%, respectively, compared with W1, and there was no significant difference between W2 and W3. Under the same water deficit condition, the overall trend of leaf SPAD gradually decreased with the increase in Se concentration (except for W3), and the three Se conditions (S2.5, S5, and S10) promoted leaf SPAD under the same moisture (except for S10W2). Under severe drought stress, the leaf SPAD of the S2.5 significantly increased by 4.2% compared with that of the S0; under the condition of sufficient water supply, the leaf SPAD of the S5 significantly increased by 4.4% compared with the S0, indicating that Se concentrations at 2.5 mg·L^−1^ and 5 mg·L^−1^ increased leaf SPAD under all three water deficit conditions.

### 2.4. Correlation Analysis of Indicators and TOPSIS Comprehensive Evaluation

#### 2.4.1. Correlation Analysis of Indicators

Table 2 shows the correlation analysis between various indicators of leaves: MDA, as an important indicator with a monitoring role, showed a negative correlation between all indicators related to photosynthesis and a positive correlation with the three osmoregulatory substances. Proline reached a significant correlation with MDA content (*p* < 0.05); the decrease in MDA content reflects a reduction in the degree of damage to crops.

The activities of GSH-PX and CAT in antioxidant enzymes were highly significant and negatively correlated with proline content (*p* < 0.01). The enhancement of GSH-PX and CAT activities under drought stress was accompanied by a decrease in the degree of lipolysis of plant cell membranes and an enhancement of photosynthesis in plants, which was highly significant and positively correlated with all indicators of photosynthesis as well as with biomass (*p* < 0.01), and was accompanied by a gradual decrease in the content of osmoregulatory substances, which showed a highly significant negative correlation with proline content (*p* < 0.01).

Combined with Figure 3a, it can be seen that SOD activity increased under drought stress; plant cell water decreased cell solutes and increased soluble sugar content, which showed a highly significant positive correlation with soluble sugar content (*p* < 0.01).

Yield per plant [21] (Appendix A) was significantly positively correlated with GSH-PX and CAT activities, negatively correlated with osmoregulatory substances proline and soluble protein, and significantly positively correlated with photosynthetic characteristics.

#### 2.4.2. TOPSIS-Based Analysis of Relationships between Treatments and Comprehensive Evaluation

The weights of the evaluation indexes related to oxidative damage and photosynthetic characteristics are shown in Table 3. The weights of proline, Pn, Ci, Tr, and yield per plant were higher, which showed that these indexes are closely related to the oxidative damage and photosynthetic characteristics. The TOPSIS comprehensive evaluation method was used to estimate the comprehensive performance of Se on the oxidative damage and photosynthetic characteristics under drought stress. The GSH-PX, CAT, POD, SOD, MDA, proline, soluble sugar, soluble proteins, Pn, Gs, Ci, Tr, SPAD, and yield of a single plant were comprehensively analyzed (Table 4), and the order of magnitude of the synthesis scores of the TOPSIS analysis was S5W3, S2.5W3, S10W3, S2.5W2, S0W3, S5W2, S10W2, S0W2, S5W1, S2.5W1, S10W1, and S0W1. It can be seen that the S5W3 had the best overall evaluation, followed by the S2.5W3.

## 3. Discussion

The production and removal of reactive oxygen species (ROS) in the body are in dynamic balance during normal plant growth [22]. However, when plants are subjected to abiotic stress, it leads to an excessive accumulation of ROS, elevated H_2_O_2_ and O^2−^ content, and disruption of cellular homeostasis, which induces oxidative damage possibly resulting in plasma membrane damage or even leading to cell death [23]. In the occurrence of drought stress, the interconversion and accumulation of reactive oxygen species such as hydroxide ions (OH^−^), superoxide anions (O^2−^), hydroxyl radicals (-OH), and hydrogen peroxide (H_2_O_2_) are induced to activate the antioxidant system of the plant, which contributes to the up-regulation or down-regulation of antioxidant enzyme activities [24,25] in order to mitigate the effects of oxidative stress in the plant. Meanwhile, plants will maintain cell expansion pressure by accumulating osmoregulatory substances (osmoregulation is an adaptive response of crops to tolerate and resist drought stress [23]) to reduce cellular osmotic potential and regulate crop tolerance to low water potential [26,27].

The MDA content reflects the extent of oxidative damage in plant cells when subjected to abiotic stresses. In the present study, we showed that heavy drought stress significantly increased the MDA content and increased the lipidation of plant cell membranes, as well as activated the plant’s own antioxidant capacity. Ali et al. [22] showed that drought stress greatly increased leaf MDA content as well as antioxidant enzyme activities such as SOD. Related studies on ROS production showed that drought stress increased the level of reactive oxygen species, and antioxidant enzyme activities and osmoregulatory substance contents were significantly increased [28,29,30]. In this study, as the degree of water deficit increased, the activities of SOD and POD increased, and the contents of the osmoregulators proline and soluble sugar were significantly increased to alleviate the damage to the membrane system.

Se is involved in the synthesis of GSH-PX, so the antioxidant effect of Se is realized through GSH-PX, and Se may be the triggering factor that initiates specific genes related to the synthesis of this enzyme, which resists the effects of the adversity factors by increasing the enzyme content and improving the enzyme activity [31]. It has been shown that SeA increased the activity of antioxidant enzymes, thereby reducing the oxidative damage caused by drought stress [9,32]. And, the study of Rady et al. [9] also found that SeA had a positive effect on the health of leaf tissues by increasing the relative water content and regulating the water status of leaves, mainly due to the increase in osmoregulatory substances and antioxidants, which reduces the amount of H_2_O_2_ and O^2−^ content to come, thus protecting the cell membranes from the effects of lipid peroxidation. In this study, SeA increased leaf GSH-PX activity and also significantly reduced MDA content, indicating that SeA reduced the degree of cell damage, but SeA at too large a concentration (10 mg·L^−1^) had a diminished ability to mitigate cell damage. SOD, POD, and CAT, as important antioxidants, play important roles in scavenging reactive oxygen radicals, resisting oxidative damage, and regulating the redox state of the organism [33,34]. Among them, SOD, as the first line of defense for plants to resist the superoxide anion (O^2−^), can have a disproportionate reaction of O^2−^ to produce oxygen (O_2_) and hydrogen peroxide (H_2_O_2_). CAT decomposes the H_2_O_2_ in the body to produce H_2_O and O_2_, and POD further catalyzes the decomposition of peroxides using a variety of reducing agents in the plant body as electron acceptors, preventing the accumulation of reactive oxygen species and limiting free radicals from initiating membrane lipid peroxidation, thus avoiding or reducing oxidative damage in plant cells [35,36]. In this study, SeA under drought stress increased CAT activity and decreased SOD and POD activities; SeA under adequate water supply conditions increased POD activity and decreased leaf MDA content under different levels of water deficit, which showed that SeA alleviated the oxidative damage of drought stress on plants. While this study lacked the observation of reactive oxygen species levels, the next study will also observe H_2_O_2_ and O^2−^, which can get the amount of free radicals scavenged by Se and also determine the drought resistance of Se on plants more directly.

In the study on osmoregulatory genes, when a crop is subjected to drought stress, the cell maintains cytoplasmic osmotic pressure through the accumulation of osmoregulatory substances, such as proline and betaine, to ensure that the cell normally absorbs water to maintain normal physiological functions [37]. The results of the present study showed that proline content increased dramatically with increasing degree of drought stress, and the osmotic regulator proline content under severe drought stress was 9.7 times higher than that under adequate water supply conditions. Rady et al. [9] showed that a water deficit produced more proline and soluble sugars compared to normal irrigation, and that the crop maintains drought stress tolerance by regulating cell expansion pressure, and Se regulates net osmotic pressure accumulation to maintain plant water status under drought conditions [38]. With the increase in spraying concentration, the proline content gradually increased, especially under severe drought stress. The proline content of the treatment sprayed with a high concentration of Se was slightly higher than that of the S0. It is possible that, with the increase in Se concentration, Se toxicity was further induced in the plants subjected to drought stress [19], thus making the increase in the proline content an important indicator to reflect the degree of plant stress.

The experiment of Ma et al. [39] investigated the ecological adaptation strategies of different types of *mallow plants* by determining the content of soluble sugars, an osmotic regulator substance, in different types of mallows. The results showed that the soluble sugar content of the strongly arid species was much larger than that of the dry species, which in turn was much larger than that of the raw species, and that the accumulation of soluble sugar was an adaptive response of the strongly arid and dry species to regulate the osmotic potential of the cells and protect the water balance. Therefore, the content of soluble sugars in leaves was significantly increased under drought stress in this study [20], and as SeA alleviated the drought stress, the accumulation of soluble sugars gradually decreased, and the difference reached a significant level (*p* < 0.05) under severe drought stress. It indicated that the interaction between SeA and water deficit resulted in a significantly greater reduction in soluble sugars under severe drought stress than in adequate water supply conditions.

“The father of plant selenoproteins”, Hongfeng Bai, found that the Se component of plants mainly exists in the form of selenoamino acids, that is, in the form of selenoproteins. Under drought stress, due to the short growth of plants, the same amount of Se is applied to the actual operation, so with the increase in drought, the greater the Se content of the leaves of the plant, the larger the selenoprotein content is. Se is involved in protein metabolism and is converted to various amino acids such as selenocysteine (Se-Cys), selenomethionine (Se-Met), selenocystine (Cy-Se), and other amino acids soon after entering the plant body in the form of inorganic Se [40]; this may be due to the fact that Se was present as a free selenoamino acid in the plant body when it was sprayed with a low concentration of Se. The mass fraction of selenoproteins in the body is relatively small, and it exists in the form of selenoproteins when sprayed at increased concentrations. The study of Ying Hu et al. [41] showed that the low amount of Se decreased the mass fraction of protein, but with the increase in Se, the mass fraction of protein gradually increased, and some studies have shown that SeA has a tendency to promote the soluble protein content [42].

Se will first activate its own antioxidant enzyme activity under drought stress, while increasing osmoregulatory substances to maintain cell expansion pressure to adapt to drought stress. Se is involved in the synthesis of GSH-PX, and the antioxidant enzyme system resistance was improved by SeA to scavenge free radicals to avoid oxidative damage in plants; thus, SeA alleviated the oxidative damage of drought stress on plant cells.

Drought stress affects cell expansion pressure, resulting in lower osmotic pressure and elevated osmoregulatory substances; thus, plants reduce the sensitivity of stomata in response to drought stress in order to maintain the stability of physiological activities such as moderate stomatal conductance and transpiration, improve cellular water retention, and thus adapt to drought-stressed environments [9,23]. The drought stress treatment in this study was a prolonged drought with reduced soil moisture, which increased the difficulty of water uptake by the plant root system, which in turn affected the water status of the plant, reducing leaf water potential and leaf water content, increasing cellular solute concentration, and reducing leaf Tr. SeA increased water retention in plant tissues by adding organic and inorganic osmoprotectants that increased water uptake in the dense and activated root system, thus not reducing the Tr [9]. The upper epidermal stomata of leaves are fewer and almost all exist in the lower epidermis, and the stomata in the lower epidermis of leaves under drought stress are more crumpled, deformed, and depressed under the surrounding epidermal cells, and the morphology of stomatal structure is altered [43]. The decrease in stomatal density and the decrease in leaf Gs reduced stomatal-induced transpiration, leading to a decrease in leaf Pn, and therefore a decrease in leaf photosynthesis under drought stress. The study of Rady et al. [9] showed that leaves exposed to air and drought stress made chloroplasts damaged, whereas optimal SeA reduced chloroplast damage, increased chlorophyll content, and activated photosynthesis by regulating components of the plant defense system; thus, SeA increased leaf Pn and Gs. 

There is a close correlation between leaf SPAD and chlorophyll content, which is one of the necessary components for photosynthesis in plants and can indirectly reflect the strength of photosynthetic capacity [44]. In contrast, this study showed that leaf SPAD showed a trend of increasing and then decreasing as the degree of water deficit decreased, that leaf SPAD under mild drought stress was the largest, and that mild drought stress promoted the uptake of nitrogen by the plant, which is an important component of chlorophyll [45]. In the study by Li [46] on water–nitrogen interactions, a moderate increase in irrigation increased leaf SPAD and tended to increase with increasing irrigation levels, and the increase in nitrogen fertilizer significantly increased leaf SPAD. Therefore, SeA under mild drought stress could promote SPAD.

The results of the correlation analysis among indicators showed that with the occurrence of drought stress, plant cell membrane lipidation increased and photosynthesis weakened, while the content of osmoregulatory substances gradually increased to protect the plant from drought stress. Additionally, SeA increased GSH-PX activity, which, according to the results of correlation analysis, showed a highly significant correlation with photosynthesis in all cases. The results of the TOPSIS comprehensive analysis showed that the combined performance of spraying low and medium concentrations of Se was better than that of the control, indicating that SeA alleviated the oxidative damage of drought stress on plant cells and promoted photosynthesis in plants.

## 4. Materials and Methods

### 4.1. Overview of the Test Site

The experiment was conducted from March to June 2022 in a solar greenhouse at Xinxiang Comprehensive Experimental Base of the Chinese Academy of Agricultural Sciences (N35°9′, E113°47′, altitude 74 m). The climate of the test area is warm temperate continental monsoon climate, with an average annual rainfall of 580 mm, an average annual evaporation of 2000 mm, an average multi-year temperature of 14.1 °C, sunshine hours of 2398.8 h, and a frost-free period of 201 d. The total area of the solar greenhouse used in the experiment was 510 m^2^ (60 m × 8.5 m), facing south. The outermost layer of the greenhouse was covered with quilts of 5 cm thickness for heat preservation, covered with a layer of 0.2 mm non-drip polyethylene film, and embedded with heat preservation materials in the walls of the side and back walls. The experiment was carried out in pots, in which the soil was selected from the cultivated layer (0~20 cm) of a large field; the texture was sandy loam, the soil capacity was 1.40 g·cm^−3^, and the water-holding field capacity was 23.02% (mass water content). The soil mass ratios of alkaline-dissolved nitrogen, quick potash, and quick phosphorus were 77.35, 486.6, and 16.03 mg·kg^−1^, respectively. The pH value was 8.65, the EC was 161.96 µS·cm^−1^, and organic matter mass fraction was 15.19 g·kg^−1^.

### 4.2. Experimental Design

The pots used in the experiment were 30 cm in diameter and 50 cm in height. The soil for the test was air-dried and mixed through a 2 mm mesh sieve, and then loaded into the pots in 3 layers, and tamped for 1 time when the pots were loaded for about 15 cm. Then, the basal fertilizer was mixed and applied into the 3rd layer, and loaded to the upper mouth of the pots at a point of 2–3 cm from the upper mouth of the pots. The mass of the air-dried soil loaded into each pot was 45.7 kg. During the test, per kg of dry soil, the application of N, P_2_O_5_, and K_2_O fertilizers was 0.13, 0.08, and 0.13 g, respectively, and the base fertilizer was mainly ordinary quick-acting compound fertilizers (N:P_2_O_5_:K_2_O = 17:15:15). The application of ordinary quick-acting compound fertilizers was calculated to be 13.95 g on the basis of 40% of the total amount of nitrogen fertilizer. Phosphorus fertilizer was applied as base fertilizer (calcium superphosphate, P_2_O_5_: 14%). The remaining 60% of nitrogen fertilizer (urea, N: 46%) and insufficient potash (potassium sulfate, K_2_O: 50%) were divided into three portions, respectively, and applied with water at the beginning of fruit expansion in each spike. The application rate of each treatment was the same. The test variety is Jingfan 404, transplanted in five leaves and one heart (16 March 2022) to the pot, each pot planting 1 plant, fruiting 3 spikes to leave 3 pieces of top leaves after topping. The experiment ended on 25 June.

**Figure 8:**
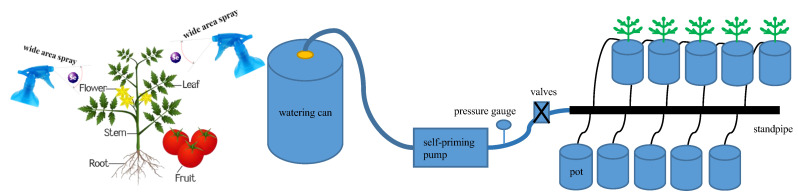
Schematic diagram of SeA and irrigation system.

Based on the results of the 2021 trial [47], in which exogenous Se spraying concentrations were 0, 5, and 10 mg·L^−1^, respectively, spraying low concentrations of exogenous Se had a significant promotion effect on growth. In this experiment, two factors, foliar exogenous Se (Na_2_SeO_3_) spraying concentration S (control concentration range: ±0.1 mg·L^−1^) and irrigation level W (control water amount range: ±5%) were set up, respectively. Four foliar sprays of exogenous Se, at concentrations of 0 (S0), 2.5 (S2.5), 5 (S5), and 10 mg·L^−1^ (S10), were set to be uniformly sprayed on the leaves of the plants during the flowering and fruiting period at two intervals of 20 d (10 and 30 d after flowering), with each spray being applied until dripping on the surface of the leaves was produced (Figure 8) [8]. The mass of the Se solution was 80 mL per plant per spray, and S0 was sprayed with water as a control. During the Se spraying process, the soil surface was covered with a waterproof plastic sheet to prevent Na_2_SeO_3_ solution from dripping into the soil and affecting the experimental results. Three irrigation levels were set at each Se spraying concentration (the lower limit of irrigation was controlled as 50% of field water-holding capacity, 65% of field water-holding capacity, and 80% of field water-holding capacity [7], which were recorded as W1, W2, and W3, respectively, and the irrigation quota was 2 L), and a total of 12 treatments were used in a complete combinatorial experimental design with 20 pots in each treatment to satisfy the need for destructive sampling for a total of 240 pots. In order to accurately control the irrigation water volume in each treatment, each treatment was individually supplied with water by a drip irrigation system consisting of an irrigation bucket, a small self-priming pump (Wugufengdeng, DP-1436), a pressure gauge, a valve, capillaries, and drip arrows equipped with flow regulators (Figure 8), with the rated flow rate of the waterer being 2 L·h^−1^, and the operating pressure being 0.12 MPa. Add the required amount of irrigation water into the irrigation bucket, start the self-priming pump, adjust the working pressure to 0.12 MPa, and inject the irrigation water into each basin through the capillary tube connected to the drip arrow until all the irrigation water in the bucket is used up.

Three representative pots of plants were selected for each treatment from 07:30 to 08:30 each day, and a small, tracked traveling crane with an electronic crane scale (accuracy of 20 g) was used to weigh the pot mass and calculate the soil water deficit degree for irrigation control.

### 4.3. Major Observation Programs and Methods

#### 4.3.1. Soil Base Indicators

(1) Soil capacity and field water-holding capacity: before the start of the experiment, the soil capacity and field water-holding capacity of the test field soil were determined by the ring knife method; since all the test soils were 20 cm topsoil, 0–20 cm ring knives were taken at different locations and repeated 6 times.
(2) Soil base nutrients: before transplanting, air-dried and sieved test soils were taken for measuring total nitrogen, quick-acting nitrogen, phosphorus, potassium, pH value, conductivity, and organic matter; total nitrogen was measured by AA3 flow analyzer [48], fast-acting potassium was measured by flame photometry, fast-acting nitrogen was measured by alkaline dissolution and diffusion method, fast-acting phosphorus was measured by sodium bicarbonate leaching method, pH value was measured by potentiometric method, electrical conductivity was measured by DDS-307 conductivity meter method, and organic matter was measured by colorimetric method.

#### 4.3.2. Physiological Indicators

(1) Photosynthesis and other physiological indexes: a sunny and cloudless day was selected at the fruit ripening stage, and the leaf photosynthetic parameters were measured using the photosynthesis measurement system LI-6400 (LI-COR Inc., Lincoln, NE, USA) from 9:00–11:00 a.m.; the plants were selected to have the fourth well-grown functional leaf from top to bottom, and three representative plants were randomly selected for measurement in each treatment. The measurement items included photosynthetic physiological indexes such as net Pn, Tr, Gs, and Ci.
(2) SPAD: at the same time as determining photosynthetic indexes, the SPAD value of functional leaves was determined by using a SPAD analyzer, and three plants with roughly the same growth were labeled for each treatment, and one leaf was labeled for each plant, and three points were measured and averaged.

#### 4.3.3. Leaf Enzyme Activities and Osmoregulatory Substances

On the 15th day after the application of Se, 3 plants were measured in 3 replicates each time, and the functional leaves of the same leaf position of the 5th to 8th plants were taken from the top to the bottom of the plants at 8:00–11:00 a.m. The leaves were washed with deionized water to clean the impurities on the leaves, then wiped dry, wrapped in tin foil, frozen in liquid nitrogen quickly, brought back to the laboratory, placed in an ultra-low-temperature refrigerator at −80 °C, stored there, and were used to determine the soluble proteins, proline, and soluble sugar content. Soluble protein content was determined by Caumas Brilliant Blue G-250 staining method [49]; proline content was determined by the acid-hydrated ninhydrin method; and soluble sugar content was determined by the anthrone method [9]. CAT activity was determined by potassium permanganate titration; POD activity was determined by guaiacol method [49]; SOD activity was determined by nitrogen blue tetrakisovanes (NBT) photoreduction assay; GSH-PX activity was determined by micro assay [1]; and MDA content was determined by colorimetric method of thiobarbituric acid (TBA) [9].

### 4.4. Data Processing

The statistical software DPS 9.01 data processing system was used to analyze the ANOVA of the experimental data, and Duncan’s new complex polarity method was applied for multiple comparisons and significance tests of differences between the experimental treatments, and plotted with Origin 2018.

## 5. Conclusions

This study investigated the effects of Se on oxidative damage and photosynthesis under drought stress, and the results showed that drought stress caused the lipolysis of plant cell membranes and activated its oxidative stress response. Se induced the occurrence of different enzyme reactions to maintain normal photosynthesis in cells, which in turn mitigated the adverse effects caused by drought stress. In this study, SeA modes were screened out for irrigation applications to save water and stabilize yield (S5W3 and S2.5W3), which provides a certain theoretical basis and technical support for the cultivation of Se-enriched tomatoes in the future. 

## Figures and Tables

**Table 1:** Net photosynthetic rate, stomatal conductance, intercellular carbon dioxide concentration, and transpiration rate of leaves under different treatments.

Processing Number	Pn (μmol CO_2_ m^−2^ s^−1^)	Gs (mol H_2_O m^−2^ s^−1^)	Ci (μmol mol^−1^)	Tr (mmol H_2_O m^−2^ s^−1^)
S0W1	15.88 ± 1.08 d	0.08 ± 0.01 e	35.67 ± 16.86 c	3.83 ± 0.39 e
S2.5W1	15.90 ± 0.31 d	0.07 ± 0.00 e	34.32 ± 3.55 c	3.53 ± 0.10 e
S5W1	16.04 ± 0.29 d	0.08 ± 0.00 e	50.13 ± 11.98 c	3.79 ± 0.04 e
S10W1	14.67 ± 1.16 d	0.07 ± 0.01 e	41.24 ± 10.76 c	3.67 ± 0.28 e
S0W2	22.83 ± 0.52 c	0.33 ± 0.03 cd	264.4 ± 7.27 ab	7.52 ± 0.28 d
S2.5W2	26.02 ± 0.18 b	0.39 ± 0.02 bcd	266.29 ± 4.04 ab	9.58 ± 0.28 c
S5W2	28.50 ± 0.42 a	0.39 ± 0.01 bcd	252.96 ± 3.18 b	10.28 ± 0.34 c
S10W2	23.71 ± 0.25 c	0.30 ± 0.02 d	240.65 ± 11.87 b	9.27 ± 0.30 c
S0W3	26.69 ± 0.38 b	0.42 ± 0.08 bc	263.85 ± 16.77 ab	11.45 ± 0.59 b
S2.5W3	29.58 ± 0.41 a	0.62 ± 0.07 a	293.87 ± 8.08 a	13.38 ± 0.60 a
S5W3	30.00 ± 0.5 a	0.46 ± 0.02 b	265.33 ± 6.84 ab	12.85 ± 0.36 a
S10W3	26.74 ± 0.31 b	0.37 ± 0.03 bcd	250.40 ± 10.31 b	11.67 ± 0.32 b
W	0.0001 **	0.0001 **	0.0001 **	0.0001 **
S	0.0001 **	0.0023 **	0.1353 ns	0.0003 **
S × W	0.0039 **	0.031 *	0.2357 ns	0.0159 *

Note: “ns” indicates means not significant (*p* > 0.05), * indicates significance at *p* < 0.05, and ** indicates significance at *p* < 0.01. Pn: net photosynthetic rate; Gs: stomatal conductance; Ci: intercellular CO_2_ concentration; Tr: transpiration rate; and means were separated according to Duncan’s multiple range test. The means with the same small case letters are statistically non-significant.

**Table 2:** Correlation analysis of indicators of leaves.

Correlation Coefficient	GSH-PX	CAT	SOD	POD	MDA	Proline	Soluble Sugar	Soluble Proteins	Pn	Gs	Ci	Tr	SPAD	Yield per Plant
GSH-PX	1	0.72 **	−0.26	−0.53	−0.3	−0.84 **	−0.49	−0.46	0.89 **	0.83 **	0.86 **	0.93 **	0.74 **	0.93 **
CAT	0.72 **	1	−0.39	−0.61 *	−0.62 *	−0.79 **	−0.54	−0.3	0.77 **	0.71 **	0.74 **	0.71 **	0.80 **	0.66 *
SOD	−0.26	−0.39	1	0.21	0.55	0.17	0.78 **	−0.29	−0.24	−0.28	−0.16	−0.28	−0.25	−0.27
POD	−0.53	−0.61 *	0.21	1	0.60 *	0.73 **	0.48	0.41	−0.61 *	−0.48	−0.63 *	−0.53	−0.76 **	−0.48
MDA	−0.3	−0.62 *	0.55	0.60 *	1	0.57 *	0.54	0.24	−0.42	−0.35	−0.31	−0.34	−0.65 *	−0.3
Proline	−0.84 **	−0.79 **	0.17	0.73 **	0.57 *	1	0.37	0.74 **	−0.92 **	−0.88 **	−0.92 **	−0.89 **	−0.94 **	−0.88 **
Soluble Sugar	−0.49	−0.54	0.78 **	0.48	0.54	0.37	1	−0.28	−0.29	−0.26	−0.28	−0.35	−0.33	−0.35
Soluble proteins	−0.46	−0.3	−0.29	0.41	0.24	0.74 **	−0.28	1	−0.69 **	−0.71 **	−0.70 **	−0.65 *	−0.67 *	−0.66 *
Pn	0.89 **	0.77 **	−0.24	−0.61 *	−0.42	−0.92 **	−0.29	−0.69 **	1	0.96 **	0.95 **	0.98 **	0.90 **	0.95 **
Gs	0.83 **	0.71 **	−0.28	−0.48	−0.35	−0.88 **	−0.26	−0.71 **	0.96 **	1	0.94 **	0.96 **	0.84 **	0.95 **
Ci	0.86 **	0.74 **	−0.16	−0.63 *	−0.31	−0.92 **	−0.28	−0.70 **	0.95 **	0.94 **	1	0.93 **	0.89 **	0.92 **
Tr	0.93 **	0.71 **	−0.28	−0.53	−0.34	−0.89 **	−0.35	−0.65 *	0.98 **	0.96 **	0.93 **	1	0.82 **	0.99 **
SPAD	0.74 **	0.80 **	−0.25	−0.76 **	−0.65 *	−0.94 **	−0.33	−0.67 *	0.90 **	0.84 **	0.89 **	0.82 **	1	0.77 **
Yield per plant	0.93 **	0.66 *	−0.27	−0.48	−0.3	−0.88 **	−0.35	−0.66 *	0.95 **	0.95 **	0.92 **	0.99 **	0.77 **	1

Note: “*” means significant (*p* ≤ 0.05); “**” means extremely significant (*p* ≤ 0.01).

**Table 3:** Weights of evaluation indexes related to oxidative damage and photosynthetic characteristics.

Assessment Criteria	GSH-PX	CAT	SOD	MDA	Proline	Soluble Sugar	Pn	Gs	Ci	Tr	SPAD	Yield per Plant
Weight	0.0807	0.0628	0.0717	0.0734	0.0940	0.0625	0.0938	0.0814	0.1039	0.0947	0.0836	0.0976

**Table 4:** TOPSIS analysis to evaluate the comprehensive performance of oxidative damage and photosynthetic characteristics.

Processing Number	D+	D−	C	Sorting Results
S0W1	0.329	0.065	0.165	12
S2.5W1	0.255	0.159	0.384	10
S5W1	0.249	0.181	0.421	9
S10W1	0.299	0.135	0.311	11
S0W2	0.162	0.205	0.559	8
S2.5W2	0.096	0.272	0.738	4
S5W2	0.098	0.265	0.729	6
S10W2	0.139	0.218	0.611	7
S0W3	0.098	0.265	0.73	5
S2.5W3	0.088	0.307	0.778	2
S5W3	0.075	0.292	0.795	1
S10W3	0.091	0.271	0.749	3

## Data Availability

The authors confirm that the data supporting the findings of this study are available within the article.

## Appendix A

**Table A1:** Yield in different treatments.

Processing Number	Number of Fruits per Plant/N	Average Fruit Mass per Fruit/g	Yield per Plant/kg
S0W1	7.11 ± 0.42 c	22.85 ± 0.47 ef	0.162 ± 0.010 e
S2.5W1	7.33 ± 0.33 c	26.82 ± 0.48 e	0.197 ± 0.010 e
S5W1	7.89 ± 0.51 c	23.61 ± 0.72 ef	0.185 ± 0.010 e
S10W1	8.11 ± 0.42 c	22.18 ± 0.57 f	0.179 ± 0.008 e
S0W2	10.33 ± 0.41 b	81.67 ± 1.60 d	0.844 ± 0.036 d
S2.5W2	10.67 ± 0.37 ab	89.41 ± 1.36 c	0.955 ± 0.041 c
S5W2	11.44 ± 0.47 ab	82.44 ± 1.31 d	0.940 ± 0.034 c
S10W2	11.11 ± 0.35 ab	81.04 ± 1.13 d	0.899 ± 0.027 cd
S0W3	11.00 ± 0.37 ab	129.92 ± 2.07 ab	1.427 ± 0.046 b
S2.5W3	11.67 ± 0.17 a	133.95 ± 2.20 a	1.562 ± 0.028 a
S5W3	11.56 ± 0.18 a	131.50 ± 2.09 ab	1.519 ± 0.028 a
S10W3	11.00 ± 0.29 ab	128.49 ± 2.36 b	1.413 ± 0.042 b
W	0.0001 ***	0.0001 ***	0.0001 ***
S	0.0584 ns	0.0001 ***	0.0005 ***
S × W	0.5629 ns	0.7580 ns	0.2946 ns

Note: Different lowercase letters show that mean values are significantly different from one another at *p* ≤ 0.05. “ns” means not significant (*p* > 0.05); “***” means extremely significant (*p* ≤ 0.001).
